# In Memoriam: Edward “Ned” Lally

**DOI:** 10.3390/pathogens9030177

**Published:** 2020-03-01

**Authors:** Nataliya Balashova

**Affiliations:** Department of Basic and Translational Sciences, School of Dental Medicine, University of Pennsylvania, Philadelphia, PA 19104, USA; natbal@upenn.edu

On February 11, 2019, we lost a colleague and friend Dr. Edward “Ned” Lally. He was an internationally recognized leader in research on the periodontal pathogen *Aggregatibacter actinomycetemcomitans*.

Ned succeeded in his professional career as a clinical pathologist, scientist and educator. He received his B.S. degree in 1966 and his D.M.D. degree in 1968 from the University of Pittsburgh. He was a resident in both the United States Naval Hospital and the Department of Pathology at the Hospital of the University of Pennsylvania. Ned received his Certificate in Oral Pathology in 1973 and earned a Ph.D. in Immunology in 1978 from the University of Pennsylvania. He then joined the Department of Pathology at the University of Pennsylvania, School of Dental Medicine, where he had grown to the rank of Professor with tenure. In addition to his academic career, Ned distinguished himself in service to the United States Navy and Naval Reserve.


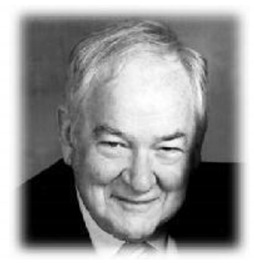


In early years, Ned’s research was focused on understanding mucosal immunity. In his practice he met young patients with an aggressive form of periodontal disease and investigated familial aggregation of the disease. Ned had a longstanding interest in the field of microbial pathogenesis and the role of *A. actinomycetemcomitans* in the development of aggressive periodontitis. He was especially intrigued to unravel the mechanism of action of an RTX toxin, LtxA, an immune cell killer. In the 1980s, Ned made his seminal observation that LtxA interacted with β2 integrin receptor LFA-1 on the surface of immune cells. He believed this was the most significant of his contributions related to the field. Following his study, β2 integrin receptors were reported for RTX toxins from other bacteria. In later years, Ned successfully employed various immunological, biophysical, biochemical and molecular techniques for the characterization of the toxin interaction with the host cell membrane. Ned found that the interaction of LtxA with immune cells is both complex and multifaceted, involving both β2 integrin LFA-1 and cell membrane cholesterol. His results provide new insight into the mechanism by which the RTX group of bacterial toxins kill cells. Ned published over 90 peer-reviewed manuscripts and successfully maintained continuous NIH funding.

Ned will be remembered not just for his scientific contributions, but also as a great charismatic teacher for young researchers. Outside his professional career, Ned was a devoted husband, father and grandfather. Personally, I will always remember him as one of the best friends I have ever met in my professional life.

